# CT-Based Sarcopenic Nomogram for Predicting Progressive Disease in Advanced Non-Small-Cell Lung Cancer

**DOI:** 10.3389/fonc.2021.643941

**Published:** 2021-10-08

**Authors:** Xiaoping Yi, Qiurong Chen, Jingying Yang, Dengke Jiang, Liping Zhu, Haipeng Liu, Peipei Pang, Feiyue Zeng, Changyong Chen, Guanghui Gong, Hongling Yin, Bin Li, Bihong T. Chen

**Affiliations:** ^1^ Department of Radiology, Xiangya Hospital, Central South University, Changsha, China; ^2^ National Clinical Research Center for Geriatric Disorders (Xiangya Hospital), Central South University, Changsha, China; ^3^ Hunan Key Laboratory of Skin Cancer and Psoriasis, Xiangya Hospital, Changsha, China; ^4^ Hunan Engineering Research Center of Skin Health and Disease, Xiangya Hospital, Changsha, China; ^5^ State Key Laboratory of Ophthalmology, Zhongshan Ophthalmic Center, Sun Yat-sen University, Guangzhou, China; ^6^ Xiangya School of Medicine, Central South University, Changsha, China; ^7^ Department of Urology, Sun Yat-sen Memorial Hospital, Sun Yat-sen University, Guangzhou, China; ^8^ Department of Radiology, the Second Affiliated Hospital of Hunan University of Chinese Medicine, Changsha, China; ^9^ Department of Pharmaceuticals Diagnosis, GE Healthcare, Hangzhou, China; ^10^ Department of Pathology, Xiangya Hospital, Central South University, Changsha, China; ^11^ Department of Oncology, Xiangya Hospital, Central South University, Changsha, China; ^12^ Department of Diagnostic Radiology, City of Hope National Medical Center, Duarte, CA, United States

**Keywords:** sarcopenia, body composition, platinum-based chemotherapy, progressive disease, non-small-cell lung cancer

## Abstract

**Background:**

It is prudent to identify the risk for progressive disease (PD) in patients with non-small-cell lung cancer (NSCLC) who undergo platinum-based chemotherapy. The present study aimed to develop a CT imaging-based sarcopenic nomogram for predicting the risk of PD prior to chemotherapy treatment.

**Methods:**

We retrospectively enrolled patients with NSCLC who underwent platinum-based chemotherapy. Imaging-based body composition parameters such as skeletal muscle index (SMI) for assessment of sarcopenia were obtained from pre-chemotherapy chest CT images at the level of the eleventh thoracic vertebral body (T11). Sarcopenic nomogram was constructed using multivariate logistic regression and performance of the nomogram was evaluated by discrimination, calibration curve, and decision curve.

**Results:**

Sixty (14.7%) of the 408 patients in the study cohort developed PD during chemotherapy. The prediction nomogram for developing PD achieved a moderate efficiency with an area under the curve (AUC) of 0.75 (95% CI: 0.69-0.80) for the training cohort, and 0.76 (95%CI: 0.68-0.84) for the validation cohort, as well as a good performance of consistence (bootstrap for training cohort: 0.75 ± 0.02; validation cohort: 0.74 ± 0.06). Favorable clinical application was observed in the decision curve analysis.

**Conclusion:**

Our CT-based sarcopenic nomogram showed the potential for an individualized prediction of progression for patients with NSCLC receiving platinum-based chemotherapy.

## Introduction

Lung cancer is the most common cancer and one of the leading causes of cancer death (1). Non-small-cell lung cancer (NSCLC) account for about 80% of all lung cancer cases (2) and advanced NSCLC patients (Stage III and IV) account for approximately 65% of all NSCLC patients upon diagnosis (3). Platinum-based chemotherapy is considered the first-line treatment strategy according to the NCCN guidelines (4). However, treatment response to this chemotherapy regimen is varied. Although a large proportion of patients achieves remission, some patients will develop progressive disease (PD) during treatment. Patients with PD not only do not benefit from chemotherapy, but also suffer from severe chemotherapy toxicity. Additionally, those patients are deprived of a chance for selecting a more effective treatment such as the new targeted therapies. Previous studies have identified several potential biomarkers such as specific genes, blood ctDNA, and circulating tumor cells etc. associated with treatment response and prognosis (5–7). However, these biomarkers are insufficient for predicting PD. There remains an unmet need for identifying patients with NSCLC at high risk for PD while undergoing platinum-based chemotherapy.

Computed tomography (CT) is the most commonly used imaging modality for evaluating NSCLC and it has been mostly widely used method for cancer diagnosis and for accessing treatment response of patients with NSCLC undergoing chemotherapy. Although CT imaging characteristics such as tumor volume and morphology are known to be associated with aggressiveness and outcome of NSCLC, these imaging features alone are not sufficient for assessing chemotherapy resistance in patients with NSCLC before treatment. New imaging methods are needed for predicting the risk of patients who may be resistant to chemotherapy.

Sarcopenia is a syndrome characterized by a loss of skeletal muscle mass and physical function such as muscle strength or physical performance, and is associated with a high risk of adverse consequences including falls, fractures, disability, poor quality of life, and increased use of hospital services (8). Sarcopenia often precedes frailty, which is a phenotype implying depletion of physiologic reserves (9). Prior studies have reported a high prevalence of sarcopenia in cancer patients especially those at advanced stage, including patients with lung cancer (10), gastric cancer, advanced hepatocellular carcinoma and metastatic renal cell carcinoma (11). Sarcopenia has also been shown to be associated with cancer diagnosis, treatment response (12, 13) and prognosis (14). For example, a study comparing CT-quantified muscle and fat distribution before and after chemotherapy showed that early detection and prevention of sarcopenia may potentially reduce chemotherapy-induced toxicity and improve outcome (15). However, there is limited information about whether sarcopenia can be used to predict chemotherapy resistance in patients with NSCLC receiving platinum-based chemotherapy.

Abdominal CT and MRI are commonly used for assessing body composition including skeletal muscle mass in the lumbar region (16) but they are not always obtained for initial imaging of NSCLC. On the other hand, chest CT is routinely acquired in clinical practice and is readily available in patients with lung cancer. Therefore, it is prudent to evaluate body composition and sarcopenia on pre-chemotherapy chest CT images.

The present study aimed to assess chest CT imaging-based sarcopenic parameters for predicting the risk of PD in NSCLC patients undergo platinum-based chemotherapy. The purpose of this study was to develop a machine learning model incorporating the chest CT-based sarcopenic parameters for identifying those who had higher risk for PD, thus assisting clinicians in selecting optimal treatment strategies for patients with NSCLC.

## Materials and Methods

### Study Cohort

This study was approved by our institutional review board (IRB: 20200401076). The written informed consents were waived due to the retrospective nature of this study.

**Figure 1:**
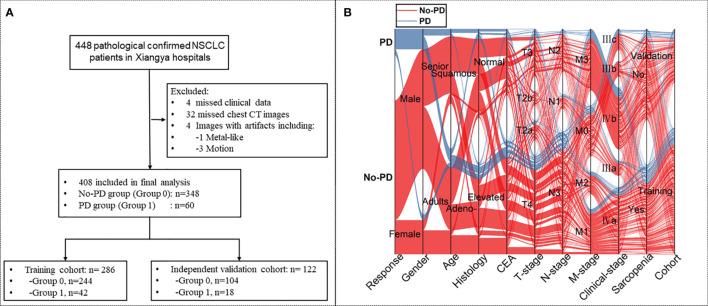
**(A)** Flow-chart demonstrating the recruiting process for patients with non-small-cell lung cancer (NSCLC) with or without progressive disease (PD). **(B)** Sankey diagram showing the scmap cluster projection of the outcome of the cases, gender, age, pathological type, expression of carcinoembryonic antigen (CEA), cancer staging, body mass index (BMI), sarcopenic status, as well as the cohort information in the predictive modeling.

Consecutive patients with pathologically confirmed NSCLC at advanced stages (Stage III/IV) and with a history of standard platinum-based chemotherapy were retrospectively identified through searching our institutional medical database from May 2011 and March 2018. NSCLC staging was determined according to the NCCN guidelines (4). Pre-chemotherapy clinicopathological data and the initial chest CT images prior to chemotherapy were obtained from the data base and the imaging system by two study radiologists (QC, JY). The retrieved laboratory data included the neutrophil to lymphocyte ratio (NLR), lymphocyte to monocyte ratio (LMR), platelet-to-lymphocyte ratio (PLR) and monocyte to lymphocyte ratio (MLR). Patients with incomplete data or had other chemotherapy were excluded from this study. Details of the patient recruiting process are shown in **Figure 1**.

### Re-Analysis of Pathology Specimen

For each patient, all pathological slides of the primary NSCLC tumors were re-analyzed by two pathologists specialized in lung cancer (GG and HY, with 6 and 25 years of experience, respectively). Consensus was reached by discussion when discrepancy occurred.

### Treatment, Follow-Up and Outcome Parameters

The primary endpoint of the study was PD according to RESIST 1.1 criterion (17). The secondary endpoint was overall survival (OS). All patients received one standard chemotherapy course (6 cycles, every 3 weeks/cycle). All patients had follow-up assessments, and the follow-up period was determined from the first day of diagnosis to the day of endpoints. OS was defined as the time from diagnosis to death due to any cause or to the last follow-up for the survivors.

### CT Image Acquisition and Body Composition Measurement

All patients underwent a non-enhanced chest CT scan prior to chemotherapy on one of the three CT scanners, i.e., a 16-MDCT (Brilliance 16, Philipps), a 64-MDCT (SOMATOM Definition, Siemens), and a 320-MDCT (Aquilion ONE, Toshiba Medical Systems) scanner. All chest CT images were retrieved from the same Picture Archiving and Communication Systems (PACS, Carestrem, Canada), and were transferred to the same external workstation (Leonardo; Siemens Medical Solutions, Forchheim, Germany). All axial CT images were reconstructed to a thickness of 1 mm.

One axial CT image was selected at the level of the eleventh thoracic vertebra level (T11) for measurement of skeletal muscle from each CT scan. In addition, a second axial CT image at the porta hepatic level was selected for measurement of visceral fat. Skin, visceral organs, bone, and spinal canal were excluded manually in the selected axial images. Body composition features of skeletal muscle was measured as previously reported (18). The chest wall and back muscles including the psoas, paraspinal, transversus abdominis, rectus abdominis, internal oblique muscles, and external oblique muscles were manually segmented on the selected axial image at the T11 level for each scan. The area of the chest wall and back muscles were calculated by using the area of pixels with threshold attenuation between -30 and 150 Hounsfield units (Hu) in the areas of interest. In addition, all segmentation results from the threshold segmentation were checked and corrected manually on the post-processing workstation by the two radiologists (FZ and DJ). When there were discrepancies in image evaluation, they reached a consensus through discussion.

Total body fat area, visceral fat area, subcutaneous fat area, muscle fat area (MFA) and skeletal muscle area (SMA) were measured manually on the selected axial images on a workstation (Advantage Windows workstation 4.6, GE Healthcare, Milwaukee, Wisconsin, USA). Skeletal muscle index (SMI) at the T11 level was calculated as the total skeletal muscle area (SMA) (cm^2^) divided by the square vertical length (m^2^) from T1 thru T10. Muscle fat index (MFI) was calculated as the area of muscle fat (MFA) divided by the square vertical length (m^2^) from T1 thru T10. Visceral or subcutaneous fat index (VFI or SFI) was calculated as the area of visceral or subcutaneous fat (cm^2^) at the porta hepatis level divided by the square vertical length (m^2^) from T1 thru T10.

**Figure 2:**
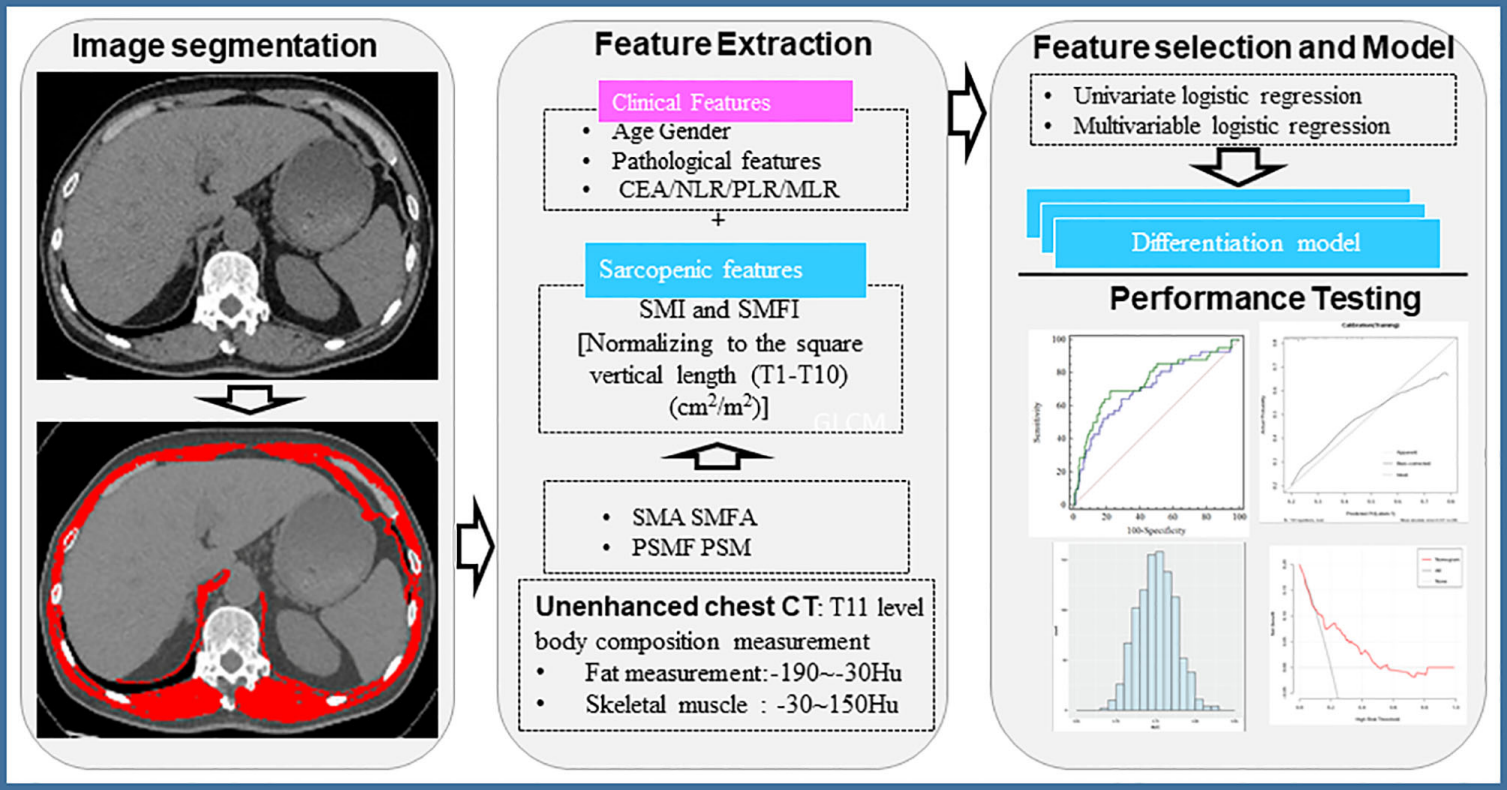
Workflow of chest CT image-based evaluation of body composition. CEA, Carcinoembryonic antigen; NLR, Neutrophil to lymphocyte ratio; PLR, Platelet-to-Lymphocyte ratio; MLR, Monocyte to Lymphocyte ratio; SMI, Skeletal muscle index; SMFI, subcutaneous muscle fat index; SMA, skeletal muscle area; SMFA, subcutaneous muscle fat area.

Sarcopenia was defined using cut‐off values determined specifically for cancer patients (18–20). Since there were no specific cut-off values for sarcopenia for muscle mass at the T11 level, we used a cut-off value which was determined based on tertiles stratified by sex (18). CT imaging analysis, and predictive modeling were presented in **Figure 2**.

### Reproducibility of Body Composition Feature Measurement

Two radiologists, (reader 1, FZ, with 11 years of experience in CT imaging; reader 2, DJ, with 18 years of experience in CT imaging) performed independent segmentations and body composition measurements on 30 randomly chosen patients’ chest CT scans. The inter-observer (reader 1 versus reader 2) and intra-observer (reader 1 twice, 4 weeks apart) correlation coefficient (ICC) values were evaluated. Our results demonstrated satisfactory inter- and intra-observer reproducibility of the body composition features measured by the two radiologists. The inter-observer ICCs of body composition features between reader 1 (first time) and reader 2 ranged from 0.971 to 1.000. The intra-observer ICC of reader 1’s two performances ranged from 0.981 to 1.000. As a result, the body compositions measured by reader 1 were used in all subsequent analysis.

### Feature Selection

Feature selection was performed using the least absolute shrinkage and selection operator (LASSO) method. The LASSO method sufficiently reduced the number of features obtained from the demographic, clinicopathological, laboratory and CT imaging-based body composition data in our analysis and it helped to select the most predictive features from the primary data. In addition, we also carried out the redundancy analysis on these features, and the results showed that the correlation coefficients between the features extracted from each sequence were less than 0.8. We used the variance inflation factor (VIF) to measure multicollinearity problems between multivariable. The VIF values in all selected features were less than 5, indicating that there was no redundancy and multicollinearity.

### Development of an Individualized Prediction Model

Multivariate logistic regression analysis was used to determine predictors associated with the risk for PD. To reduce overfitting, only the selected factors based on multivariate analysis were used to build the models. The nomogram for predicting the risk of PD was constructed with sarcopenic parameters obtained on the chest CT images.

### Performance Data and Clinical Application of the Nomogram

Calibration of the nomogram was evaluated by calibration curves (Hosmer-Lemeshow H test), and the diagnostic efficiency was quantified using the receiver operating characteristic (ROC) curve and the area under the curve (AUC). To estimate the prediction error, we further tested the proposed model using a 1000-iteration bootstrap analysis in both the training cohort and the validation cohort. Decision curve analysis was performed to evaluate the clinical usefulness of the nomogram in the validation cohort.

### Statistical Analysis

All statistical analysis was implemented with R software (http://www.Rproject.org). Multivariate binary logistic regression, nomograms and calibration plots were done with the “rms” package. Decision curve was performed with the “rmda” package. The “survival” package was used for survival analysis. The statistical significant levels were all two-sided with statistical significance set at 0.05. For the quantitative features, the Wilcoxon rank-sum test was used. For the qualitative features, Chi-square test or Fisher’s exact test was used to test differences between the groups.

Continuous and categorical variables were presented as median (min-max) and n (%), respectively. We used the Mann-Whitney U test, Fisher’s exact test to compare differences between the PD group and no-PD group where appropriate. To explore the risk factors associated with PD, univariate and multivariate logistic regression models were used. We excluded variables from the univariate analysis if between-group differences were not significant, and if the number of events was too small to calculate the odds ratios.

A logistic regression analysis was applied to identify independent predictors for PD, and the individualized prediction model was developed by multivariate logistic analysis. The calibration curves were used to assess the performance of the nomogram. The diagnose performance of established models was quantified by the ROC curve and the AUC. Decision curve analysis was conducted to validate the clinical usefulness of the nomogram by quantifying the net benefits at different threshold probabilities.

## Result

### Clinicopathological Characteristics of the Patients

A total of 408 patients with advanced NSCLC were included in this study, and 60 of them developed PD during chemotherapy. All patients were randomly assigned to either the training cohort (n=286) or the validation cohort (n=122) at a 2: 1 ratio (**Figure 1**).

Patients’ characteristics and the comparison between the PD group and the no-PD group were presented in **Table 1**. Significant differences between the PD group and the no-PD group were found for age (especially male patients), body height, lymphocyte to monocyte ratio (LMR), and incidence of thoracic sarcopenia with P<0.05 (**Table 1**). Except for a marginal difference regarding the SMI value of female patients, all variables in the training cohort and the validation cohort showed no statistical difference (P>0.5) indicating a reasonable separation of these two groups.

**Table 1:** Demographic, clinicopathological, and CT body composition features of patients with advanced non-small cell lung cancer.

Characteristic	Total (n = 408)	Patients with PD (n = 60)	Patients with no-PD (n = 348)	P Value	Training Cohort (n = 286)	Validation Cohort (n = 122)	P Value
Demographics, clinical and pathological characteristics							
Gender				0.836			0.566
Male	317	46 (76.7)	271(77.9)	•••••••	220 (76.9)	97 (79.5)	•••••••
Female	91	14 (23.3)	77(22.1)	•••••••	66 (23.1)	25 (20.5)	•••••••
Age (y)^#^							
Male	57.9 (48.4-67.4)	55.2 (43.1-63.0)	58 (49.8-66.2)	0.034^*^	57.8 (47.7-67.9)	58.0 (49.9-66.1)	0.724
Female	52.3 (42.2-62.4)	48.1 (38.0-58.3)	53.1 (43.1-63.0)	0.094	52.1 (42.0-62.1)	52.9 (42.5-63.3)	0.885
Both	56.6 (46.7-66.6)	53.5 (44.0-63.1)	57.2 (47.3-67.1)	0.008^**^	56.5 (46.1-66.9)	57.0 (48.1-65.8)	0.672
Height (cm)	164.2 (157.3-171.1)	166.9 (160.7-173.1)	163.8 (154.9-170.7)	0.001^**^	163.8 (156.8-170.9)	165.1 (158.6-171.7)	0.076
BMI							
Obesity	89 (21.8)	14 (23.3)	75 (21.6)	0.758	57 (19.9)	32 (26.2)	0.158
Pathological type				0.067			0.399
Adenocarcinoma	207 (50.7)	37 (61.7)	170 (48.9)	•••••••	149 (52.1)	58 (47.5)	•••••••
Squamous cell carcinoma	201 (49.3)	23 (38.3)	178 (51.1)	•••••••	137 (47.9)	64 (52.5)	•••••••
TNM stage							
T (primary tumor)				0.276			0.060
T2a	45 (11.0)	7 (11.7)	38 (10.9)	•••••••	30 (10.5)	15 (12.3)	•••••••
T2b	32 (7.8)	1 (1.7)	31 (8.9)	•••••••	29 (10.1)	3 (2.5)	•••••••
T3	107 (26.2)	18 (30.0)	89 (25.6)	•••••••	71 (24.8)	36 (29.5)	•••••••
T4	224 (54.9)	34 (56.7)	190 (54.6)	•••••••	156 (54.5)	68 (55.7)	•••••••
N (regional lymph nodes)				0.661			0.778
N1	32 (7.8)	6 (10.0)	27 (7.8)	•••••••	22 (7.7)	11 (9.0)	•••••••
N2	97 (23.8)	11 (18.3)	86 (24.7)	•••••••	66 (23.1)	31 (25.4)	•••••••
N3	279 (68.4)	43 (71.7)	235 (67.5)	•••••••	198 (69.2)	80 (65.6)	•••••••
M (distant metastases)				0.440			0.360
M0	116 (28.4)	19 (31.7)	97 (27.9)	•••••••	76 (26.6)	40 (32.8)	•••••••
M1a	102 (25.0)	10 (16.7)	92 (26.4)	•••••••	74 (25.9)	28 (23.0)	•••••••
M1b	51 (12.5)	9 (15.0)	42 (12.1)	•••••••	40 (14.0)	11 (9.0)	•••••••
M1c	139 (34.1)	22 (36.7)	117 (33.6)	•••••••	96 (33.6)	43 (35.2)	•••••••
Clinical stage				0.197			0.127
IIIa	34 (8.3)	6 (10.0)	28 (8.0)	•••••••	23 (8.0)	11 (9.0)	•••••••
IIIb	35 (8.6)	2 (3.3)	33 (9.5)	•••••••	27 (9.4)	8 (6.6)	•••••••
IIIc	47 (11.5)	11 (18.3)	36 (10.3)	•••••••	26 (9.1)	21 (17.2)	•••••••
IVa	152 (37.3)	19 (31.7)	133 (38.2)	•••••••	113 (39.5)	39 (32.0)	•••••••
IVb	140 (34.3)	22 (36.7)	118 (33.9)	•••••••	97 (33.9)	43 (35.2)	•••••••
Laboratory findings							
Blood routine examination							
NLR	3.2 (0.0-48.3)	3.6 (0.2-40.0)	3.1(0.0-48.3)	0.071	3.3 (0.0-48.3)	3.0 (0.9-46.0)	0.204
LMR	2.8 (0.5-252.5)	2.4 (0.6-252.5)	2.8 (0.5-252.5)	0.048^*^	2.6 (0.46-252.5)	2.8(0.6-252.5)	0.128
PLR	160.0 (2.7-847.1)	169.8 (11.2-847.1)	159.6 (2.7-650.0)	0.241	162.0 (2.7-670.0)	154.8 (8.1-847.1)	0.658
CEA							
CEA (>5)	258 (63.2)	41(68.3)	217 (62.4)	0.375	174 (60.8)	84 (68.9)	0.124
CT Imaging features							
Body composition features							
SMI							
Male	42.9 (18.4-62.4)	38.8 (29.9-52.7)	43.2 (18.4-62.4)	0.481	42.9 (19.5-62.4)	42.9 (18.4-59.1)	0.920
Female	34.5 (22.7-58.5)	33.6 (25.2-41.9)	34.6 (22.7-58.5)	<0.001^**^	33.7 (22.9-58.5)	35.4 (22.7-43.7)	0.043^*^
MFI	1.8 (-16.5-19.5)	1.6 (0.1-6.7)	1.9 (-16.5-19.5)	0.341	1.8 (-16.5-19.5)	2.1 (-0.1-7.0)	0.131
Thoracic sarcopenia	202 (49.5)	42 (70.0)	160 (46.0)	0.001^**^	146 (51.0)	56 (45.9)	0.341

Unless otherwise indicated, data are numbers of patients, and data in parentheses are percentages.

^#^ age was presented as media (minimum~ maximum).

^*^ indicates P<0.05. ^**^ indicates P<0.01.

PD, progressive disease; BMI, body mass index; NLR, Neutrophil to lymphocyte ratio; LMR, Lymphocyte to monocyte ratio; PLR, platelet-to-lymphocyte ratio; CEA, Carcinoembryonic Antigen Test; SMI, skeletal muscle index; MFI, muscle fat index.

When compared to the no-PD group, the PD group showed significantly higher incidence of sarcopenia (P<0.001) and significantly lower SMI value for female patients (P<0.001) (**Table 1**).

### Risk Factors for PD

**Figure 3:**
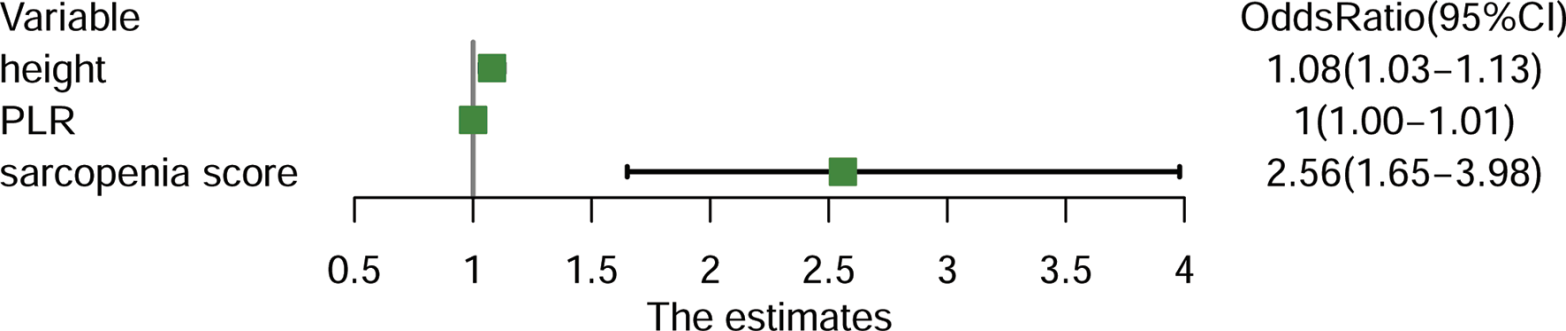
Forest plot demonstrating the independent predictors for progressive disease (PD) for patients with advanced non-small-cell lung cancer (NSCLC). PLR, Platelet-to-Lymphocyte ratio.

A sarcopenic score was generated based on the three parameters, i.e., SMI, age and gender. In univariate analysis, sarcopenic score, sarcopenia, PLR, NLR, and body height were associated with higher risk of PD (all P<0.05). Subsequent multivariate logistic regression analysis identified the higher sarcopenic score, body height, and PLR being the independent predictors for higher risk of PD (**Figure 3**).

### Model Performance

**Figure 4:**
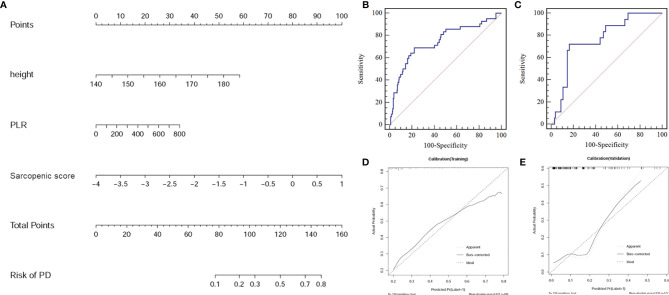
**(A)** Sarcopenic nomogram constructed in the training cohort with the sarcopenic score, platelet-to-lymphocyte ratio (PLR), and body height. **(B)** The receiver operating characteristic (ROC) curve for the training cohort. **(C)** The ROC curve for the validation cohort. **(D)** Calibration curve of the sarcopenic nomogram in the training cohort. **(E)** Calibration curve of the sarcopenic nomogram in the validation cohort presenting the calibration of the predictive model for the risk of transition to progressive disease (PD).

The final model constructed with sarcopenic score, PLR, and body height achieved a moderate efficiency with an AUC of 0.75 (95% CI:0.69-0.80) for the training cohort, and 0.76 (95%CI: 0.68-0.84) for the validation cohort, respectively. The model also had a good performance of consistence (bootstrap, Training: 0.75 ± 0.02; Validation: 0.74 ± 0.06) (**Figures 4A**–**C**).

The calibration curve of the nomogram for the risk of PD showed good agreement between prediction and observation in both cohorts (**Figures 4D, E**). Using the Hosmer-Lemeshow H test, a non-significant statistical data for both the training cohort (P =0.848) and the validation cohort (P =0.527) was obtained, suggesting no departure from perfect fit during modeling.

**Figure 5:**
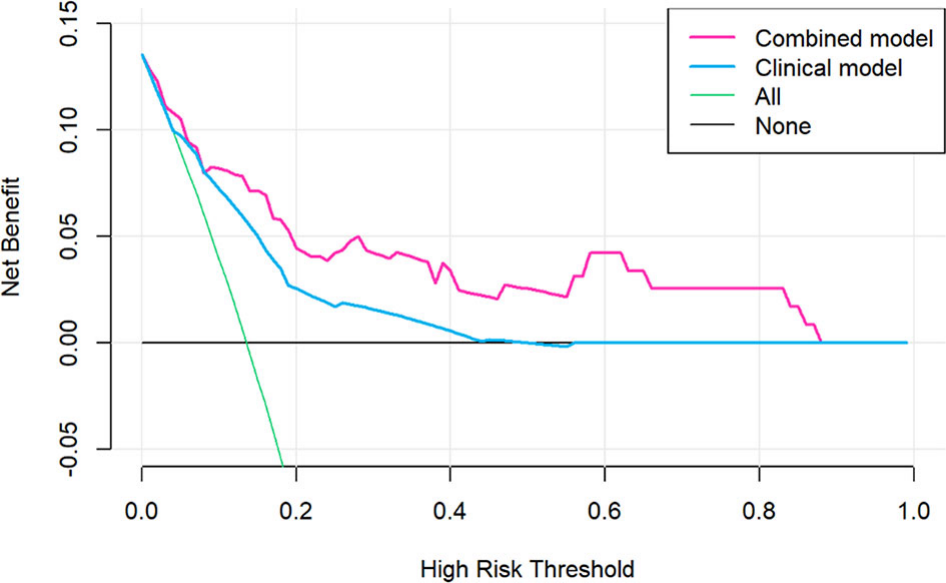
Decision curve analysis for the sarcopenic nomogram. The y-axis measures the net benefit. The red line represents the sarcopenic nomogram. The grey line represents the assumption that no patients had risk for progressive disease (PD) and the thin black line represented all patients would develop PD.

The decision curve analysis for the nomogram was presented in **Figure 5**. If the threshold probability of a patient or doctor was greater than 15%, then using the nomogram to predict PD may add more benefit than either the diagnose-all-patients scheme or the diagnose-none scheme.

## Discussion

In this study, we assessed sarcopenia on pretreatment chest CT images of patients with advanced NSCLC and developed a sarcopenic nomogram to predict the patients with higher risk of PD while undergoing chemotherapy. Our predictive model combining CT sarcopenic parameters and clinical features efficiently predicted the risk of PD. Our study highlighted the importance of pre-chemotherapy body composition assessment for predicting treatment response and chemotherapy sensitivity in patients with advanced NSCLC.

Currently, chest CT is not done for body composition measurement, but rather a routine tool to evaluate lung lesions. In general, chest CT scans do not extend down to the L3 level, which is the commonly used level for estimating skeleton muscle mass (21). We therefore measured chest body composition at the T11 level (22). Our study showed a new chest approach for body composition measurement for patients who only had chest CT available during the initial imaging evaluation. Our approach may help to avoid the additional radiation and cost for acquiring abdominal CT to cover the more commonly used L3 level for body composition assessment (21).

Prior studies have reported the clinical implication of sarcopenia in various diseases including lung cancer (23). There is significant association between sarcopenia and dismal outcome (24), longer hospital stays, more complications and worse prognosis (16). However, the underlying pathological basis for the association between sarcopenia and outcome of platinum-based chemotherapy in NSCLC patients remains unclear. Since body composition measurements may reflect metabolism of muscle, we speculate that patients with sarcopenia may have abnormally high metabolic activity due to the aggressive tumor which may lead to diminished skeletal muscle and worse outcome (25). Our speculation about metabolic derangement as the potential pathological basis was supported by literature. For instance, patients with cancer and sarcopenia have shown to have abnormal muscle protein metabolism, decreased food intake and lack of physical activity, further exacerbating abnormal metabolism in these patients and leading to treatment failure (26). In addition, chemotherapy may induce muscle wasting, resulting in further deterioration of sarcopenia (26). Furthermore, patients with sarcopenia may have decreased quality of life (27) and there is a lack of adherence to treatment, which ultimately affects the treatment effect (28). Taken together, this process may result in a vicious cycle in patients with sarcopenia and cancer, leading to further decrease of treatment response to chemotherapy.

Sarcopenia has been shown to be associated with frailty (29, 30), and may contribute to an increased risk of PD (31). Sarcopenia is usually accompanied by increasing fat mass, worsening chemotherapy toxicity, worsening outcomes and decreasing overall survival in cancer patients (32). We therefore speculate that sarcopenia in our patients with advanced NSCLC indicate a worse physical status such as frailty, which may make them less responsive to chemotherapy, and more susceptible to chemotherapy toxicity. These may lead to higher risk of PD.

The robust performance of our prediction model may be due to inclusion of important clinical and laboratory values in the model such as the body height and the ratio of platelet to lymphocyte (PLR). For example, body height has been shown to be a risk factor for poor prognosis (33), and an increased risk of future illness for some disorders such as ischemic stroke (33, 34). Previous studies have demonstrated a positive correlation between height and the incidence of lung cancer, and such an association may be due to certain genetic factors and biological pathways that affect adult height (35). Earlier studies have found that insulin-like growth factors (IGFs), which influence height by affecting the growth and development of somatic tissues, such as skeletal muscle and bone, could enhance cell proliferation, inhibit apoptosis and affect cell transformation in the context of carcinogenesis (36). Besides, high level of IGFs related to tumor directly supports chemotherapy resistance of cancer cells, and the blockade of IGFs significantly increases the response of chemotherapy drugs in pancreatic cancer and breast cancer (37, 38). In addition, prior studies have shown a significant association between pre-treatment PLR and outcomes in lung cancer, gastric cancer, colorectal cancer, ovarian cancer and hepatocellular cancer (39). Elevated PLR indicated activation of transcription factors, which could produce cytokines promoting tumor growth through inflammatory response and eventually lead to PD (40). Therefore, these relevant clinical and laboratory values were important for identifying the risk factors for PD.

Our study had several limitations. First, the retrospective nature of this study with data from a single center made case selection bias inevitable. Second, several confounding variables such as physical condition before treatment, nutritional status, smoking history, and exercise habits for the patients in our study may affect the treatment outcome, which were not included in our predictive modeling. Third, although our study included a relatively large number of patients with advanced NSCLC, the study cohort was still modest in size considering the high incidence of lung cancer. In addition, the clinical generalizability of the model may be in question since it was not validated with external data from other centers. The limited sample size and single-center data may affect the generalizability of our data. Large-scale prospective multicenter study is needed to validate our results. Lastly, we recognize the limitation of our imbalanced sample size for each group with only a small number of patients in our cohort having progressive disease. Mathematical methods such as the synthetic minority oversampling technique (SMOTE) (41) should be helpful to improve learning from the imbalanced sample sizes in our analysis, which we plan to use for our future studies.

## Conclusion

Utilizing a chest CT imaging-based sarcopenic evaluation, we developed a new prognostic method to predict the risk of PD in patients with advanced NSCLC receiving platinum-based chemotherapy. Our non-invasive approach may potentially be useful in identifying patients with higher risk of PD before chemotherapy, thus assisting clinicians in selecting optimal treatment strategies for patients with advanced NSCLC.

## Data Availability Statement

The raw data supporting the conclusions of this article will be made available by the authors, without undue reservation.

## Ethics Statement

The studies involving human participants were reviewed and approved by institutional review board of Xiangya Hospital, Central South University (IRB: 20200401076). The ethics committee waived the requirement of written informed consent for participation.

## Author Contributions

XY: Conceptualization, Methodology, Writing- Original draft preparation, Investigation, Validation, Reviewing and Editing. QC: Conceptualization, Methodology, Writing- Original draft preparation, Investigation, Validation, Reviewing and Editing. JY: Conceptualization, Methodology, Writing- Original draft preparation, Investigation, Validation, Reviewing and Editing. DJ: Data curation, Investigation. LZ: Conceptualization, Methodology, Writing- Original draft preparation, Supervision, Validation, Reviewing and Editing. HL: Conceptualization, Methodology, Writing- Original draft preparation, Supervision, Validation, Reviewing and Editing. PP: Methodology, Software, Visualization. FZ: Investigation. CC: Investigation. GG: Data curation, Investigation. HY: Conceptualization, Investigation, Supervision, Validation. BL: Validation, Reviewing and Editing. BC: Methodology, Supervision, Reviewing and Editing. All authors contributed to the article and approved the final version.

## Funding

This study was partially supported in part by Natural Science Foundation of Hunan Province, P.R. China (2018JJ2641, 2019JJ40485).

## Conflict of Interest

Author PP is employed by GE healthcare.

The remaining authors declare that the research was conducted in the absence of any commercial or financial relationships that could be construed as a potential conflict of interest.

## Publisher’s Note

All claims expressed in this article are solely those of the authors and do not necessarily represent those of their affiliated organizations, or those of the publisher, the editors and the reviewers. Any product that may be evaluated in this article, or claim that may be made by its manufacturer, is not guaranteed or endorsed by the publisher.

## Abbreviations

NSCLC, Non-small-cell lung cancer; PD, progressive disease; CT, Computed tomography; LMR, lymphocyte to monocyte ratio; NLR, Neutrophil to lymphocyte ratio; PLR, platelet-to-lymphocyte ratio; SMI, skeletal muscle index.
